# RETRACTION: Amelioration of Adults' Mental Health Conditions and Symptoms Through Spiritual Energy Therapy: Randomized Controlled Trial

**DOI:** 10.1002/npr2.70099

**Published:** 2026-02-17

## Abstract

**RETRACTION**: TrivediM. K. , BrantonA. , TrivediD. , MondalS. and JanaS. , “Amelioration of Adults' Mental Health Conditions and Symptoms Through Spiritual Energy Therapy: Randomized Controlled Trial,” Neuropsychopharmacology Reports 45, no. 3 (2025): e70050, 10.1002/npr2.70050.40931401 PMC12422956

The above article, published online on 10 September 2025 in Wiley Online Library (wileyonlinelibrary.com), has been retracted by agreement between the journal Editor‐in‐Chief, Tsuyoshi Miyakawa; The Japanese Society of Neuropsychopharmacology; and John Wiley & Sons Australia, Ltd. The retraction has been agreed upon as a similar version of this article by the same authors was previously retracted by the Journal of General and Family Medicine (https://doi.org/10.1002/jgf2.773). Although revisions have been made, the article does not adequately address the previously identified concerns. In particular, issues regarding the appropriateness of the control group and the psychological questionnaire scoring method remain unresolved. Additionally, some biomarker values remain implausible without sufficient justification and claims regarding the efficacy of spiritual energy therapy continue to lack independent supporting evidence. The editors agree the results and conclusions are unreliable. The authors disagree with the retraction.

**RETRACTION**: M. K. Trivedi , A. Branton , D. Trivedi , S. Mondal and S. Jana , “Amelioration of Adults' Mental Health Conditions and Symptoms Through Spiritual Energy Therapy: Randomized Controlled Trial,” Neuropsychopharmacology Reports 45, no. 3 (2025): e70050, 10.1002/npr2.70050.40931401 PMC12422956

The above article, published online on 10 September 2025 in Wiley Online Library (wileyonlinelibrary.com), has been retracted by agreement between the journal Editor‐in‐Chief, Tsuyoshi Miyakawa; The Japanese Society of Neuropsychopharmacology; and John Wiley & Sons Australia, Ltd. The retraction has been agreed upon as a similar version of this article by the same authors was previously retracted by the Journal of General and Family Medicine (https://doi.org/10.1002/jgf2.773). Although revisions have been made, the article does not adequately address the previously identified concerns. In particular, issues regarding the appropriateness of the control group and the psychological questionnaire scoring method remain unresolved. Additionally, some biomarker values remain implausible without sufficient justification and claims regarding the efficacy of spiritual energy therapy continue to lack independent supporting evidence. The editors agree the results and conclusions are unreliable. The authors disagree with the retraction.

